# COVID-19 Vaccination Recommendations and Practices for Women of Reproductive Age by Health Care Providers — Fall DocStyles Survey, United States, 2022

**DOI:** 10.15585/mmwr.mm7239a1

**Published:** 2023-09-29

**Authors:** Mehreen Meghani, Beatriz Salvesen Von Essen, Lauren B. Zapata, Kara Polen, Romeo R. Galang, Hilda Razzaghi, Dana Meaney-Delman, Grayson Waits, Sascha Ellington

**Affiliations:** ^1^Immunization Services Division, National Center for Immunization and Respiratory Diseases, CDC; ^2^Division of Reproductive Health, National Center for Chronic Disease Prevention and Health Promotion, CDC; ^3^Division of Birth Defects and Infant Disorders, National Center on Birth Defects and Developmental Disabilities, CDC; ^4^Oak Ridge Institute for Science and Education, Oak Ridge, Tennessee; ^5^Influenza Division, National Center for Immunization and Respiratory Diseases, CDC.

**Summary**

**What is already known about this topic?**

COVID-19 vaccination is recommended for all persons ≥6 months of age. Pregnant women are at increased risk for severe COVID-19 compared with other reproductive-aged women. Health care provider (HCP) recommendations are important for increasing vaccination coverage.

**What is added by this report?**

Although most (82.9%) surveyed HCPs recommended that women of reproductive age stay up to date with COVID-19 vaccines, only 54.7% offered or administered the vaccine in their practice. HCPs were more likely to offer or administer COVID-19 vaccination on-site to pregnant patients if they also offered or administered influenza (adjusted prevalence ratio [aPR] = 5.5) and tetanus toxoid, reduced diphtheria toxoid, and acellular pertussis (Tdap) vaccines (aPR = 2.3).

**What are the implications for public health practice?**

Encouraging HCPs to recommend, offer, and administer COVID-19 vaccines along with influenza or Tdap vaccines might help reinforce vaccine confidence and increase coverage among women of reproductive age, including pregnant women.

## Abstract

Pregnant and postpartum women are at increased risk for severe illness from COVID-19 compared with nonpregnant women of reproductive age. COVID-19 vaccination is recommended for all persons ≥6 months of age. Health care providers (HCPs) have a unique opportunity to counsel women of reproductive age, including pregnant and postpartum patients, about the importance of receiving COVID-19, influenza, and tetanus toxoid, reduced diphtheria toxoid, and acellular pertussis (Tdap) vaccines. Data from the Fall 2022 DocStyles survey were analyzed to examine the prevalence of COVID-19 vaccination attitudes and practices among HCPs caring for women of reproductive age, and to determine whether providers recommended and offered or administered COVID-19 vaccines to women of reproductive age, including their pregnant patients. Overall, 82.9% of providers reported recommending COVID-19 vaccination to women of reproductive age, and 54.7% offered or administered the vaccine in their practice. Among HCPs who cared for pregnant patients, obstetrician-gynecologists were more likely to recommend COVID-19 vaccination to pregnant patients (94.2%) than were family practitioners or internists (82.1%) (adjusted prevalence ratio [aPR] = 1.1). HCPs were more likely to offer or administer COVID-19 vaccination on-site to pregnant patients if they also offered or administered influenza (aPR = 5.5) and Tdap vaccines (aPR = 2.3). Encouraging HCPs to recommend, offer, and administer the COVID-19 vaccines along with influenza or Tdap vaccines might help reinforce vaccine confidence and increase coverage among women of reproductive age, including pregnant women.

## Introduction

Pregnant and postpartum women are at increased risk for severe COVID-19–associated illness compared with nonpregnant women of reproductive age (*1*). COVID-19 vaccination* before or during pregnancy is safe and effective and reduces the risk for severe illness and adverse COVID-19–associated outcomes (*2*–*4*). Similarly, influenza^†^ and tetanus toxoid, reduced diphtheria toxoid, and acellular pertussis (Tdap)^§^ vaccines are recommended and can be safely administered during pregnancy. Health care providers (HCPs) have a unique opportunity to counsel women of reproductive age, including pregnant and postpartum patients, about the importance of receiving COVID-19, influenza, and Tdap vaccinations (*5*,*6*). Data from the Fall 2022 DocStyles survey were analyzed to examine the attitudes and practices related to COVID-19 vaccination among HCPs caring for women of reproductive age, and to ascertain whether providers recommended and offered or administered the COVID-19 vaccines to pregnant patients.

## Methods

The Fall 2022 DocStyles survey, administered during August 19–September 30, 2022, was a web-based nonprobability panel survey of U.S. HCPs^¶^ sampled from Sermo’s global medical panel.** Quotas were predetermined to reach 1,000 family practitioners and internists, 250 obstetrician-gynecologists (ob-gyns), 250 pediatricians, and 250 nurse practitioners and physician assistants. Eligible respondents practiced only in the United States, were actively seeing patients, had been practicing for ≥3 years, and provided care to women of reproductive age (female patients aged 15–49 years). Participation was voluntary, and respondents received an honorarium ranging from $55 to $65 depending on how many questions they were asked. The survey was designed to ascertain provider attitudes and practices on a broad range of health care topics, including COVID-19 vaccination for women of reproductive age and pregnant patients, and to determine whether HCPs recommended and offered or administered COVID-19, influenza, and Tdap vaccines during pregnancy.

Descriptive analyses were conducted to determine provider characteristics (age, gender, number of years in practice, primary work setting, number of patients seen per week, and percentage of patients who were pregnant during the previous year) overall and by provider type. Prevalence of COVID-19 vaccination attitudes and practices with reference to women of reproductive age overall and by selected provider characteristics were estimated, and Pearson’s chi-square tests of independence were used to identify differences among groups, with p-values <0.05 considered statistically significant. Factors associated with recommending and offering or administering COVID-19 vaccines on-site to pregnant patients were examined using binomial regression (log-linked binomial) models; provider characteristics and influenza and Tdap vaccination attitudes and practices related to pregnant patients were considered as potential covariates. In multivariable modeling, models were adjusted for the number of years in practice and provider age and gender. Data were analyzed using SAS software (version 9.4; SAS Institute). This activity was reviewed by CDC, deemed not research, and was conducted consistent with applicable federal law and CDC policy.^††^

## Results

Among 2,587 eligible HCPs, 1,752 (68%) completed the survey (Table 1). The majority of respondents (57.2%) were family practitioners or internists; ob-gyns, pediatricians, and nurse practitioners or physician assistants each accounted for 14.3% of the sample. Nearly two thirds of survey respondents (63.9%) worked in group outpatient settings and had been in practice for >10 years (63.6%); approximately one half (55.8%) were male, and 64.3% reported that 1%–10% of their patients during the previous year were pregnant. Among ob-gyns and pediatricians, 53.6% and 51.6%, respectively, were female compared with fewer than one third (31.0%) of family practitioners and internists. One half (50.8%) of ob-gyns had been practicing for >20 years compared with approximately one third (37.4%) of family practitioners or internists, 39.6% of pediatricians, and 16.4% of nurse practitioners and physician assistants.

**TABLE 1:** Characteristics of health care providers, overall and by provider type — Fall DocStyles, United States, 2022

Characteristic	Provider type, no. (%)*				
	Total N *=* 1,752	FP or internist n = 1,002	Pediatrician n = 250	Ob-gyn n = 250	NP or PA n = 250
**Age, median, yrs (range)**	**47 (25–85)**	47 (26–84)	47 (29–75)	50 (29–85)	40 (25–71)
**Gender^†^**					
Female	**761 (43.4)**	311 (31.0)	129 (51.6)	134 (53.6)	187 (74.8)
Male	**977 (55.8)**	681 (68.0)	120 (48.0)	115 (46.0)	61 (24.4)
**No. of patients seen per week**					
1–50	**265 (15.1)**	142 (14.2)	30 (12.0)	29 (11.6)	64 (25.6)
51–100	**962 (54.9)**	532 (53.1)	144 (57.6)	149 (59.6)	137 (54.8)
101–200	**407 (23.2)**	239 (23.9)	66 (26.4)	59 (23.6)	43 (17.2)
201–500	**118 (6.7)**	89 (8.9)	10 (4.0)	13 (5.2)	6 (2.4)
**Percentage of patients who were pregnant during the previous year**					
0	**214 (12.2)**	73 (7.3)	95 (38.0)	8 (3.2)	38 (15.2)
1–10	**1,126 (64.3)**	790 (78.8)	141 (56.4)	29 (11.6)	166 (66.4)
≥11	**412 (23.5)**	139 (13.9)	14 (5.6)	213 (85.2)	46 (18.4)
**No. of years practicing**					
3–10	**637 (36.4)**	356 (35.5)	73 (29.2)	66 (26.4)	142 (56.8)
11–19	**473 (27.0)**	271 (27.1)	78 (31.2)	57 (22.8)	67 (26.8)
≥20	**642 (36.6)**	375 (37.4)	99 (39.6)	127 (50.8)	41 (16.4)
**Primary work setting**					
Individual outpatient practice	**298 (17.0)**	163 (16.3)	27 (10.8)	55 (22.0)	53 (21.2)
Group outpatient practice or clinic	**1,119 (63.9)**	634 (63.3)	181 (72.4)	171 (68.4)	133 (53.2)
Inpatient practice or hospital	**335 (19.1)**	205 (20.5)	42 (16.8)	24 (9.6)	64 (25.6)
**U.S. Census Bureau region^§^**					
Northeast	**426 (24.3)**	257 (25.8)	68 (27.2)	45 (18.0)	56 (22.4)
Midwest	**383 (21.9)**	217 (21.7)	52 (20.8)	54 (21.6)	60 (24.0)
South	**565 (32.3)**	303 (30.2)	77 (30.8)	88 (35.2)	97 (38.8)
West	**378 (21.6)**	225 (22.5)	53 (21.2)	63 (25.2)	37 (14.8)

**Abbreviations:** FP = family practitioner; NP = nurse practitioner; Ob-gyn = obstetrician-gynecologist; PA = physician assistant.

* Percentages might not sum to 100 because of rounding.

^†^ Fourteen health care providers were excluded from gender-stratified analyses because when asked their gender, they did not report male or female but instead responded “prefer to self-identify”; therefore, the denominator for gender is 1,738.

^§^ https://www2.census.gov/geo/pdfs/maps-data/maps/reference/us_regdiv.pdf

Overall, 82.9% of HCPs reported recommending COVID-19 vaccination to women of reproductive age (Table 2). The percentage of providers recommending COVID-19 vaccine varied significantly by provider type, ranging from 90.8% of ob-gyns and 90.4% of pediatricians to 76.0% of nurse practitioners and physician assistants (p<0.001). Provider perceptions of the importance of women of reproductive age staying up to date with COVID-19 vaccinations also varied substantially by provider type, ranging from 80.8% of ob-gyns to 55.6% of nurse practitioners and physician assistants reporting that staying up to date was very important (p<0.001). The importance of staying up to date with COVID-19 vaccination also varied by the percentage of patients who were pregnant that providers saw during the previous year. Among providers who reported that none of their patients were pregnant, two thirds (67.8%) reported that it was very important for women of reproductive age to stay up to date compared with three quarters (75.5%) of providers who reported that ≥11% of their patients during the previous year were pregnant (p<0.05).

**TABLE 2:** Prevalence of health care provider attitudes and practices regarding COVID-19 vaccination among women of reproductive age,* overall and by health care provider characteristics — Fall DocStyles, United States, 2022

Characteristic (no. of respondents)	Survey question, no. (row %)							
	Do you recommend that women of reproductive age* get COVID-19 vaccinations?		In general, how important do you think it is for women of reproductive age to stay up to date with their COVID-19 vaccines?				Does your practice offer or administer COVID-19 vaccines on-site to women of reproductive age?	
	Yes	No	Very important	Somewhat important	Not too important	Not at all important	Yes	No
**Provider type^†^**								
**Total (1,752)**	**1,453 (82.9)**	**299 (17.1)**	**1,230 (70.2)**	**393 (22.4)**	**73 (4.2)**	**56 (3.2)**	**958 (54.7)**	**794 (45.3)**
FP or internist (1,002)	810 (80.4)	192 (19.2)	692 (69.1)	242 (24.2)	38 (3.8)	30 (3.0)	569 (56.8)	433 (43.2)
Pediatrician (250)	226 (90.4)	24 (9.6)	197 (78.8)	40 (16.0)	10 (4.0)	3 (1.2)	163 (65.2)	87 (34.8)
Ob-gyn (250)	227 (90.8)	23 (9.2)	202 (80.8)	39 (15.6)	5 (2.0)	4 (1.6)	104 (41.6)	146 (58.4)
NP or PA (250)	190 (76.0)	60 (24.0)	139 (55.6)	72 (28.8)	20 (8.0)	19 (7.6)	122 (48.8)	128 (51.2)
**No. of years in practice^§^**								
3–10 (637)	521 (81.8)	116 (18.2)	445 (69.9)	141 (22.1)	31 (4.9)	20 (3.1)	380 (60.0)	257 (40.4)
11–19 (568)	398 (84.1)	75 (15.9)	319 (67.4)	123 (26.0)	18 (3.8)	13 (2.8)	264 (55.8)	209 (44.2)
≥20 (642)	534 (83.2)	108 (16.8)	466 (72.6)	129 (20.1)	24 (3.7)	23 (3.6)	314 (48.9)	328 (51.1)
**Percentage of patients seen during previous year who were pregnant** ^¶^								
0 (214)	170 (79.4)	44 (20.6)	145 (67.8)	44 (20.6)	14 (6.5)	11 (5.1)	101 (47.2)	113 (52.8)
1–10 (1,126)	927 (82.3)	199 (17.7)	774 (68.7)	261 (23.2)	51 (4.5)	40 (3.6)	626 (55.6)	500 (44.4)
≥11 (356)	356 (86.4)	56 (13.6)	311 (75.5)	88 (21.4)	8 (1.9)	5 (1.2)	231 (56.1)	181 (43.9)
**Gender**^¶^^,^**								
Female (761)	650 (85.4)	111 (14.6)	567 (74.5)	142 (18.7)	23 (3.0)	29 (3.8)	409 (53.8)	352 (46.3)
Male (977)	792 (81.1)	185 (18.9)	655 (67.0)	245 (25.1)	50 (5.1)	27 (2.8)	544 (55.7)	433 (44.3)

**Abbreviations:** FP = family practitioner; NP = nurse practitioner; Ob-gyn = obstetrician-gynecologist; PA = physician assistant.

* Women of reproductive age were defined as female patients aged 15–49 years.

**^†^** Pearson’s chi-square tests for independence. Statistically significant (p<0.05) when compared across provider characteristic.

**^§^** Pearson’s chi-square tests for independence. Statistically significant (p<0.05) when compared across provider characteristic for the question, “Does your practice offer or administer COVID-19 vaccines on-site to women of reproductive age?”

^¶^ Pearson’s chi-square tests for independence. Statistically significant (p<0.05) when compared across provider characteristic for the question, “In general, how important do you think it is for women of reproductive age to stay up to date with their COVID-19 vaccines?”

** Fourteen health care providers were excluded from gender-stratified analyses because when asked their gender, they did not report male or female but instead responded “prefer to self-identify”; therefore, the denominator for gender is 1,738.

Among all respondents, approximately one half (54.7%) reported offering or administering COVID-19 vaccination on-site to women of reproductive age in their practice; this varied substantially by provider type, with 65.2% of pediatricians and 41.6% of ob-gyns offering or administering COVID-19 vaccine. Offering or administering COVID-19 vaccine also varied by the number of years in practice. Among providers practicing for 3–10 years, 60.0% offered or administered the vaccine compared with 48.9% of those practicing for ≥20 years (p<0.05).

Among 1,538 providers who cared for pregnant patients, most recommended all three vaccines (COVID-19: 82.9%; influenza: 89.4%; and Tdap: 78.1%) (Supplementary Figure, https://stacks.cdc.gov/view/cdc/133101). The percentage of ob-gyns who recommended COVID-19 vaccination to their pregnant patients (94.2%) was higher than that of family practitioners and internists (82.1%; aPR = 1.1) (Table 3). Recommendations for COVID-19 vaccination were more prevalent among providers who also recommended influenza vaccine (90.0%; aPR = 3.7) and Tdap vaccine (89.8%; aPR = 1.5), and among those who offered or administered the influenza (88.2%; aPR = 1.4) and Tdap (88.7%; aPR = 1.3) vaccines.

**TABLE 3:** Factors associated with recommending and offering or administering COVID-19 vaccination on-site to pregnant patients among health care providers caring for pregnant patients (N = 1,538) — Fall DocStyles, United States, 2022

Characteristic	Recommend that pregnant patients receive COVID-19 vaccine				Offer or administer COVID-19 vaccination on-site to pregnant patients			
	No. (%)		PR (95% CI)		No. (%)		PR (95% CI)	
	Yes	No	Unadjusted	Adjusted*	Yes^†^	No^†^	Unadjusted	Adjusted*
**Provider type**								
FP or internist	763 (82.1)	166 (17.9)	Ref	Ref	519 (55.9)	410 (44.1)	Ref	Ref
Pediatrician	137 (88.4)	18 (11.6)	1.1 (1.0–1.1)	1.1 (1.0–1.1)	103 (66.5)	52 (33.6)	1.2 (1.0–1.3)	1.2 (1.1–1.3)
Ob-gyn	228 (94.2)	14 (5.8)	1.1 (1.1–1.2)	1.1 (1.1–1.2)	96 (39.7)	146 (60.3)	0.7 (0.6–0.8)	0.7 (0.6–0.9)
NP or PA	147 (69.3)	65 (30.7)	0.8 (0.8–0.9)	0.9 (0.8–0.9)	104 (49.1)	108 (50.9)	0.9 (0.8–1.0)	0.8 (0.7–1.0)
**No. of years practicing**								
3–10	475 (83.6)	93 (16.4)	1.0 (1.0–1.1)	0.9 (0.9–1.0)	328 (57.8)	240 (42.3)	1.2 (1.1–1.4)	0.9 (0.8–1.1)
11–19	343 (83.9)	66 (16.1)	1.0 (1.0–1.1)	1.0 (0.9–1.0)	227 (55.5)	182 (44.5)	1.2 (1.0–1.3)	0.9 (0.8–1.1)
≥20	457 (81.5)	104 (18.5)	Ref	Ref	267 (47.6)	294 (52.4)	Ref	Ref
**Provider age, yrs**								
<50	810 (84.7)	146 (15.3)	Ref	Ref	557 (58.3)	399 (41.7)	Ref	Ref
≥50	465 (80.0)	117 (20.1)	0.9 (0.9–1.0)	0.9 (0.9–1.0)	265 (45.5)	317 (54.5)	0.8 (0.7–0.9)	0.8 (0.6–0.9)
**Provider gender^§^**								
Female	533 (84.1)	101 (15.9)	1.0 (1.0–1.1)	1.0 (0.9–1.1)	338 (53.3)	296 (46.7)	1.0 (0.9–1.1)	1.0 (0.9–1.1)
Male	731 (82.1)	159 (17.9)	Ref	Ref	477 (53.6)	413 (46.4)	Ref	Ref
**Recommend influenza vaccine to pregnant patients**								
Yes	1,236 (90.0)	139 (10.1)	3.8 (2.9–4.9)	3.7 (2.8–4.9)	773 (56.2)	602 (43.8)	1.9 (1.5–2.4)	1.8 (1.4–2.3)
No	39 (23.9)	124 (76.1)	Ref	Ref	49 (30.1)	114 (69.9)	Ref	Ref
**Recommend Tdap vaccine to pregnant patients**								
Yes	1,078 (89.8)	123 (10.2)	1.5 (1.4–1.7)	1.5 (1.4–1.7)	674 (56.1)	527 (43.9)	1.3 (1.1–1.5)	1.3 (1.1–1.4)
No	197 (58.5)	140 (41.5)	Ref	Ref	148 (43.9)	189 (56.1)	Ref	Ref
**Offer or administer influenza vaccine to pregnant patients**								
Yes	1,095 (88.2)	146 (11.8)	1.5 (1.3–1.6)	1.4 (1.3–1.6)	788 (63.5)	453 (36.5)	5.5 (4.0–7.6)	5.5 (4.0–7.6)
No	180 (60.6)	117 (39.4)	Ref	Ref	34 (11.5)	263 (88.6)	Ref	Ref
**Offer or administer Tdap vaccine to pregnant patients**								
Yes	981 (88.7)	125 (11.3)	1.3 (1.2–1.4)	1.3 (1.2–1.4)	702 (63.5)	404 (36.5)	2.3 (2.0–2.7)	2.3 (1.9–2.7)
No	294 (68.1)	138 (31.9)	Ref	Ref	120 (27.8)	312 (72.2)	Ref	Ref

**Abbreviations:** FP = family practitioner; NP = nurse practitioner; Ob-gyn = obstetrician-gynecologist; PA = physician assistant; PR = prevalence ratio; Ref = referent group; Tdap = tetanus toxoid, reduced diphtheria toxoid, and acellular pertussis vaccine.

* Adjusted for number of years practicing, provider age, and provider gender.

^†^ Percentages might not sum to 100 because of rounding.

^§^ Four health care providers were excluded from gender-stratified analyses because when asked their gender, they did not report male or female but instead responded “prefer to self-identify”; therefore, the denominator for gender is 1,534.

Most providers also offered or administered all three vaccines on-site to pregnant patients in their practice (COVID-19: 53.5%; influenza: 80.7%; and Tdap: 71.9%). (Supplementary Figure, https://stacks.cdc.gov/view/cdc/133101). However, approximately one third (39.7%) of ob-gyns offered or administered COVID-19 vaccine on-site, compared with approximately one half of family practitioners and internists (55.9%; aPR = 0.7). Providers were more likely to offer or administer COVID-19 vaccination on-site if they also recommended influenza (56.2%; aPR = 1.8) and Tdap (56.1%; aPR = 1.3) vaccines, and if they also offered or administered influenza (63.5%; aPR = 5.5) and Tdap (63.5%; aPR = 2.3) vaccinations in their practice (Table 3).

## Discussion

The Fall 2022 DocStyles survey reported that most HCPs recommend that women of reproductive age be vaccinated against COVID-19, and the percentage was highest among ob-gyns. However, one in five family practitioners and internists did not recommend COVID-19 vaccination to women of reproductive age. This finding is consistent with other surveys on provider attitudes and practices regarding vaccination, wherein ob-gyns were more likely than were other HCPs to recommend both human papillomavirus vaccine (HPV) and COVID-19 vaccines to women of reproductive age (*7*,*8*). Most providers also felt that it was very important that women of reproductive age stay up to date with COVID-19 vaccination. However, one in five providers felt that it was only somewhat important that women of reproductive age stay up to date with COVID-19 vaccination, despite evidence that these women delay vaccination or remain unvaccinated. Staying up to date with COVID-19 vaccination is especially important because vaccines and recommendations are frequently updated in order to provide optimal protection.^§§^ Staying up to date might be particularly important for pregnant and especially recently pregnant women who are at higher risk for severe COVID-19–associated illness or adverse pregnancy outcomes.

This analysis found that provider-reported recommendation for COVID-19 vaccine to pregnant patients was strongly associated with reported recommendation for influenza and Tdap vaccines. Most providers offered or administered the COVID-19 vaccines on-site, and offering or administering COVID-19 vaccine to pregnant patients was strongly associated with recommending and offering or administering influenza and Tdap vaccines. A strong provider recommendation for vaccination has been shown to be effective in improving acceptance of HPV (*9*) and COVID-19 vaccines (*10*). As COVID-19 vaccine availability in primary care settings increases, and as more providers are tasked with offering or administering COVID-19, influenza, and Tdap vaccines, provider recommendations will continue to play an important role in motivating vaccination acceptance among women of reproductive age, especially to those who are pregnant. Previous studies on vaccination coverage among pregnant patients have found that influenza, Tdap, and COVID-19 vaccination coverage remains highest among women who report receiving a provider recommendation or offer for vaccination (*6*,*10*). HCPs are among the most trusted sources for information on vaccines, and provider recommendation or offer of vaccination is a strong predictor of vaccination (*6*,*10*). HCPs should be encouraged to recommend and offer or administer COVID-19 vaccine to women of reproductive age. All HCPs, regardless of provider type, should emphasize the importance of adhering to vaccination recommendations for women of reproductive age.

### Limitations

The findings in this report are subject to at least four limitations. First, DocStyles is a voluntary opt-in panel survey, and sampling is not population-based or random. Therefore, findings might not be generalizable to the U.S. population of HCPs. Second, survey data are self-reported, and responses might be subject to recall, social desirability, or other reporting biases. Third, data are from fall 2022 and might not reflect current provider recommendations or practices. Finally, the reasons that some HCPs might not recommend COVID-19 vaccination to women of reproductive age are unknown and were not assessed.

### Implications for Public Health Practice

COVID-19 vaccination is recommended for pregnant patients to prevent severe illness and adverse pregnancy outcomes (*10*), and HCPs are uniquely positioned to provide vaccination recommendations. Provider recommendation for vaccination is strongly associated with patient acceptance of vaccine and with vaccination coverage. Encouraging HCPs to recommend, offer, and administer COVID-19 vaccines, along with influenza or Tdap vaccines, might help reinforce vaccine confidence and increase vaccination coverage among women of reproductive age, including pregnant women. Ensuring that women of reproductive age receive these vaccines as recommended is critical to reduce the incidence of these diseases and their associated complications among pregnant women and newborns.

## Footnotes

*
https://www.cdc.gov/coronavirus/2019-ncov/vaccines/recommendations/pregnancy.html

†
https://www.cdc.gov/mmwr/volumes/67/rr/rr6703a1.htm?s_cid=rr6703a1_w

§
https://www.cdc.gov/mmwr/volumes/69/wr/mm6903a5.htm

¶ DocStyles is commissioned by Porter Novelli Public Services. http://styles.porternovelli.com

** Sermo’s global medical panel comprises 350,000 panelists who were verified using a double opt-in sign-up process with telephone confirmation at place of work. http://www.sermo.com

†† 45 C.F.R. part 46; 21 C.F.R. part 56; 42 U.S.C. Sect. 241(d), 5 U.S.C. Sect. 552a, 44 U.S.C. Sect. 3501 et seq.

§§
https://www.cdc.gov/coronavirus/2019-ncov/vaccines/stay-up-to-date.html
